# High-Throughput Identification of Antimicrobial Peptides from Amphibious Mudskippers

**DOI:** 10.3390/md15110364

**Published:** 2017-11-22

**Authors:** Yunhai Yi, Xinxin You, Chao Bian, Shixi Chen, Zhao Lv, Limei Qiu, Qiong Shi

**Affiliations:** 1 BGI Education Center, University of Chinese Academy of Sciences, Shenzhen 518083, China; yiyunhai@genomics.cn; 2 Shenzhen Key Lab of Marine Genomics, Guangdong Provincial Key Lab of Molecular Breeding in Marine Economic Animals, BGI Academy of Marine Sciences, BGI Marine, BGI, Shenzhen 518083, China; youxinxin@genomics.cn (X.Y.); bianchao@genomics.cn (C.B.); 3 State Key Laboratory of Marine Environmental Science, College of Ocean and Earth Sciences, Xiamen University, Xiamen 361102, China; chenshixi@xmu.edu.cn; 4 Fujian Collaborative Innovation Center for Exploitation and Utilization of Marine Biological Resources, Xiamen University, Xiamen 361102, China; 5 Key Laboratory of Experimental Marine Biology, Institute of Oceanology, Chinese Academy of Sciences, Qingdao 266071, China; 18012481396@163.com (Z.L.); qiulimei@qdio.ac.cn (L.Q.); 6 Laboratory of Aquatic Genomics, College of Life Sciences and Oceanography, Shenzhen University, Shenzhen 518060, China

**Keywords:** antimicrobial peptide (AMP), high-throughput identification, amphibious mudskipper, antimicrobial activity

## Abstract

Widespread existence of antimicrobial peptides (AMPs) has been reported in various animals with comprehensive biological activities, which is consistent with the important roles of AMPs as the first line of host defense system. However, no big-data-based analysis on AMPs from any fish species is available. In this study, we identified 507 AMP transcripts on the basis of our previously reported genomes and transcriptomes of two representative amphibious mudskippers, *Boleophthalmus pectinirostris* (BP) and *Periophthalmus magnuspinnatus* (PM). The former is predominantly aquatic with less time out of water, while the latter is primarily terrestrial with extended periods of time on land. Within these identified AMPs, 449 sequences are novel; 15 were reported in BP previously; 48 are identically overlapped between BP and PM; 94 were validated by mass spectrometry. Moreover, most AMPs presented differential tissue transcription patterns in the two mudskippers. Interestingly, we discovered two AMPs, hemoglobin β1 and amylin, with high inhibitions on *Micrococcus luteus*. In conclusion, our high-throughput screening strategy based on genomic and transcriptomic data opens an efficient pathway to discover new antimicrobial peptides for ongoing development of marine drugs.

## 1. Introduction

Antimicrobial peptides (AMPs) exist in all living creatures in nature and present the first line of host defense against infectious pathogens. Over the past decades, a diverse number of AMPs have been discovered from different organisms [1,2]. The publicly available Antimicrobial Peptide Database (APD) currently contains over 2900 AMPs from six kingdoms [3].

AMPs are short, cationic, amphipathic peptides with a broad spectrum of antimicrobial activity and multifaceted immunomodulatory functions [4,5]. Most AMPs can be classified into two main types. Some have their own genes (such as piscidin and cathelicidin), while some are derived from proteolysis of other immune related genes (such as histone, thrombin, lectin and cytokine). Recent years, many novel AMPs have been well defined, and some of them even have been widely applied in pharmaceuticals, health products and agriculture [6,7,8,9]. As the best candidates to replace antibiotics, AMPs have a bright future for applications in our daily life, especially those from marine resources [10,11,12,13]. However, their action mechanisms and phylogenetic status are still ambiguous [14,15,16].

It is a challenge to explore universal patterns of all AMPs in different species at the same time. Various families of AMPs form a synergistic, complex and sophisticated network in host defense system, and further studies are necessary to ascertain the phylogenetic relationships among them. Meanwhile, in any typical family of AMPs, many variable factors are yet to be revealed. For example, the putative antimicrobial activities are influenced by change of one single amino acid, addition or deletion of a fragment, 3D structure in harsh conditions, tail modification and oxidation [10,17,18,19]. We also know little about the distinct functions of one peptide in different tissues of the same organism or in various creatures living in diverse habitats. However, environment seems to be the major driving force for AMP diversity and functions [20]. It might be practical to look into the connection between environment or species distribution and AMPs. Fishes, the major aquatic vertebrates widely distributed all over the world, will definitely facilitate our deep investigation into this field because of their various habitats.

Mudskippers are a typical group of fish species for studying the transition of aquatic and terrestrial lifestyles. *Boleophthalmus pectinirostris* (BP or blue-spotted mudskipper) is predominantly aquatic while *Periophthalmus magnuspinnatus* (PM or giant-fin mudskipper) spends more time on land. Our previous genomic report [21] has shown that mudskippers have acquired many immune genes and experienced certain genetic changes for adaptations to the terrestrial life. However, the innate immune system of mudskippers has not been well explored. Here, we employed these big-data-based genome and transcriptome resources [21] to identify AMPs in these amphibious mudskippers, which are living in an inter-tidal transition between water and land.

Usually, novel AMPs have been identified and characterized by traditional isolation and experimental methods. Although previous studies provide valuable clues for our present project, these methods are often time-consuming, costly and hard to predict. Up to date, researches exploring all AMPs of one organism in the perspective of multi-omics are scarce. High-throughput identification of particular AMP genes will accelerate our pace of study towards evolution of living beings and supply abundant templates for us to examine for development of new drugs with high efficiency. Our present paper is the first report of high-throughput identification of AMPs by a combination of genomic, transcriptomic and proteomic data in amphibious mudskippers.

## 2. Results

### 2.1. Summary of Achieved Data

To identify potential AMPs, we employed BLAST to search against seven previously assembled genomic and transcriptomic datasets [14] (see more details in Section 4.1.) by our collected active AMPs (Table S1), on the basis of sequence similarity. Statistics of identified sequences are summarized in Table 1. In this present study, all putative sequences were named after the names of AMPs mapped or homologous to the BLASTP results of NCBI, followed by serial numbers if several were found. After manually inspecting our combined data (Tables S2 and S3), we obtained 223 and 204 putative AMP genes (Table S4) and 257 and 250 putative AMP sequences (File S1) in BP and PM respectively.

**Figure 1:**
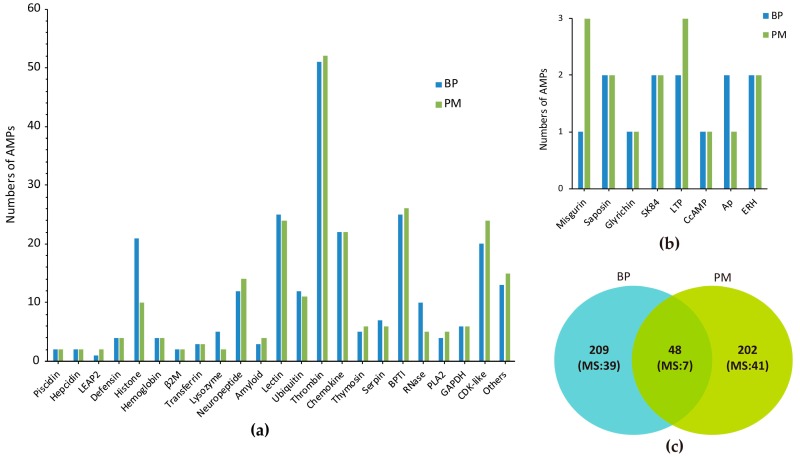
Summary of the identified AMPs from BP and PM. (**a**) Overview of different AMPs; (**b**) Types of AMPs in the “Other” group of (**a**); (**c**) Overlapping of AMPs between BP and PM. MS means to those AMPs detected by mass spectrometry.

These sequences can be classified into 30 groups (Figure 1a,b). Among them, 15 are exactly the same as previously known AMPs in the putative mature regions, including two hepcidins, one liver-expressed antimicrobial peptide 2 (LEAP2) of BP, one chrombacin (in neuropeptide group) of PM and two histones, seven ubiquitins (Ubqs), two enhancers of rudimentary (ERH, a highly conserved protein with unknown function firstly isolated from pig intestine [22]) in two mudskipper species. Additionally, 48 AMPs of BP are identical to the corresponding counterparts in PM, including 14 cyclin-dependent protein kinases (CDK-like), five histones, seven Ubqs, two ERHs, one piscidin, one Ap (an optimized *Argopecten purpuratus* antifungal peptide [23]), one misgurin (an AMP isolated from *Misgurnus anguillicaudatus* [24]), two glyceraldehyde 3-phosphate dehydrogenase (GAPDH) related proteins, one thrombin-derived C-terminal peptides (TCPs), one glyrichin, two chemokines, two thymosins, one CcAMP (an AMP from *Coridius chinensis*), seven neuropeptides and one amyloid. In total, we acquired 459 different AMPs in our currently established database (Figure 1c).

Most of these putative AMPs are related to activation of immune system. The largest group of identified AMPs is TCP, which has built a new connection between coagulation system and innate immune system [25]. Interestingly, the highly conserved cysteine-constrained structure of TCPs is also a feature of cysteine-containing AMPs [26]. In addition, bovine pancreatic trypsin inhibitor (BPTI), lectin, CDK-like, chemokine seem to be the major sources of AMPs; histone, ubiquitin and neuropeptides are also counted in. CDK-like proteins were predicted from scolopendin1 of *Scolopendra subspinipes mutilans*, which induced reactive oxygen species (ROS) accumulation in *Candida albicans* [27], suggesting their potential antifungal activity. As for histone, we found 21 copies in BP while PM had 10 only, and five of them were the same.

Ubiquitin has three forms, including Ubq/RPL40e and Ubq/RPS27a fusions as well as homopolymeric multiubiquitin protein chains. Ubq family contains several ubiquitin-like proteins (Ubql) as there are a considerable amount of UBQ domain-containing protein in vivo, such as Nedd8, Elongin B, Rub1, Parkin and ubquicidin (UBI) [28,29,30]. Neuropeptide group consists of neuropeptide YY, adrenomedullin, chromogranin A (CGA), calcitonin gene-related peptide (CGRP), vasoactive intestinal peptide (VIP), and two peptides only mapped in PM (chrombacin and catestatin). “Other” group includes eight classes of putative AMPs, which are highly similar to those isolated in various species but no general names are available (Figure 1b).

### 2.2. Top Ranked AMPs in Four Tissue Transcriptomes

**Figure 2:**
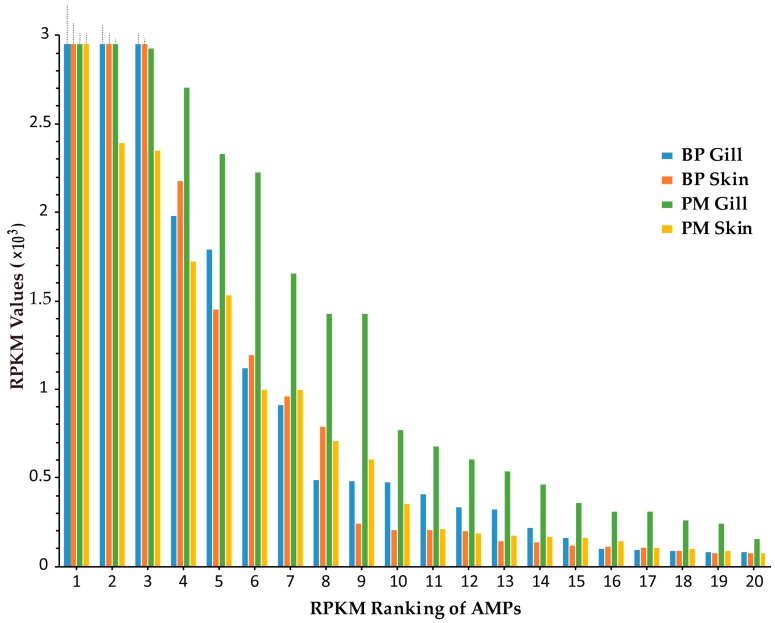
Comparison of the top 20 AMPs (with the highest RPKM values) from transcriptome datasets of gill and skin in BP and PM (Top of the first three exceedingly highly transcribed genes are delineated by dash line).

Transcription levels of putative AMP genes were illustrated by RPKM (reads per kilobase of transcript per million mapped reads) values in our previous report [21]. The results in skin and gill of two mudskippers were sorted by RPKM values (see more details in Table S4). The top 20 highly transcribed AMPs in each tissue are ranked in Figure 2. All of them were above 70 (Figure 2). The types of AMPs ranked the top 20 in four tissues are listed in Table 2. Subunits of hemoglobin (Hb) in BP gill had particularly high RPKM values (top 3: α2, 41,420; β1, 12,092; α1, 6048). They also ranked top three in BP skin (α2, 17,282; β1, 7408; α1, 4211). In PM, β2 microglobulin (β2M) and chemokine CCL4 owned rather high (top two) RPKM values (7234 and 4945, respectively) in the gill, while GAPDH1 ranked the number one (8153) in the skin. We also observed that the transcription levels in PM gill were generally higher than those in other three tissues, suggesting their potential vital roles for terrestrial adaptation.

**Figure 3:**
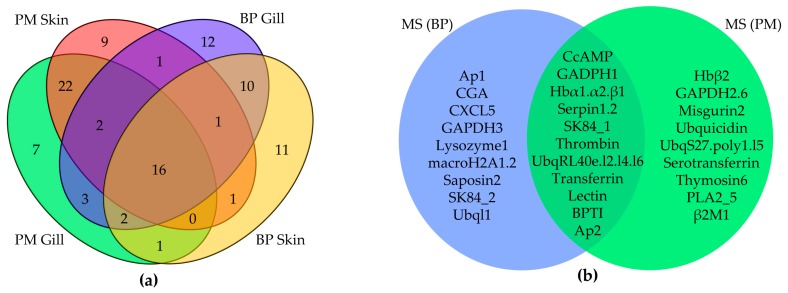
Venn diagrams of AMPs from various datasets. (**a**) Relationship of identified AMPs with RPKM values above 20 from four tissue transcriptomes; (**b**) Overlapping of AMPs detected by MS between BP and PM.

AMPs with RPKM values above 20 (gene number: BP skin, 42; BP gill, 47; PM skin, 52; PM gill, 53) were picked out from the four transcriptome datasets for comparison, and we found that 16 of them are common (Figure 3a), namely six chemokines, ERH1, GADPH1, glyrichin, H2A, three Hb subunits (α1, α2, β1), saposin2 and two Ubqs. Among these common AMPs, three Hb subunits, Ubq/RPL40e and GAPDH1 were also detected by mass spectrometry (MS) in the skin of two mudskipper species (Figure 3b). The majority of these putative AMPs are chemokines and proteases, with the remarkable capacity to kill microorganisms in vitro. Interestingly, TCPs were almost confined in the gill, while lectins were limited in the skin.

### 2.3. MS Validation

To validate the variety of AMP peptides in different tissues, we applied a shotgun ESI-MS approach (see more details in Section 4.3). A summary of these MS results in the muscle and skin of two mudskipper species is presented in Table 3. All those detected spectra of putative AMP precursors are listed in Table S5. We observed that the numbers of identified spectra and proteins in PM were more than BP. Gene ontology (GO) annotation of skin also revealed that proteins associated with catalytic activity, binding, metabolic process and cellular process were richer in PM (File S2), and each category in the skin was more abundant than in the muscle. Interestingly, in the BP muscle, CGA was the only AMP that had not been detected in the BP skin. However, in the PM muscle, all proteins were detected in the PM skin except lectin17.

It was demonstrated that the fragments of 46 putative AMPs in BP and 48 putative AMPs in PM did exist at the protein level. The overlapping relationship of detected AMPs between BP and PM is summarized in Figure 3b. After comparing the results of top AMPs in the four transcriptome datasets (Table 2), we observed that some were detected (such as TCPs, lectins and BPTIs) and some were not (like hepcidins, piscidins, defensins and CDK-like proteins). However, some absent in the top RPKM list were also detected by MS, such as CGA, Hbβ2, CcAMP and serpins (serine protease inhibitors).

### 2.4. Antimicrobial Confirmation

In our preliminary screening of 23 synthesized peptides from our deduced AMP list (Table S6), 11 were demonstrated to exhibit a significant inhibitory activity to Gram-positive *Micrococcus luteus* at a concentration of 250 μM (Figure S1), while they didn’t present antimicrobial abilities against *Aeromonas hydrophila*, *Vibrio parahaemolyticus* or *Staphylococcus aureus* (data not shown). We subsequently chose three of them to determine their minimum inhibitory concentration (MIC) since they significantly suppressed the growth of *M. luteus*.

**Figure 4:**
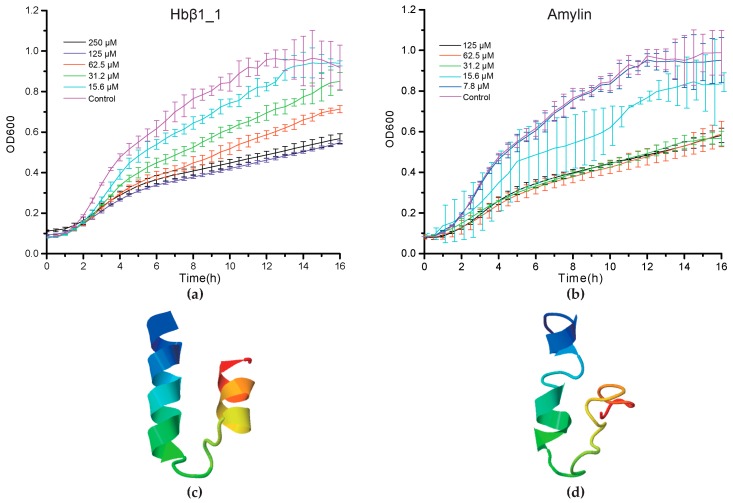
The dynamic suppression of Hbβ1 (**a**) and amylin (**b**) on the growth of *Micrococcus luteus*. Vertical bars represent the mean ± standard deviation (*n* = 3). Structural conformation model of Hbβ1 (**c**) and amylin (**d**) was predicted by I-TASSER with the highest confidence (see more details in Section 4.6).

Consequently, we found that Hbβ1_1 and amylin of BP displayed a strong antibacterial activity against *M. luteus* at a MIC < 31.2 μM and a MIC < 15.6 μM respectively (*p* < 0.05, Figure 4a,b). However, piscidin2 did not exhibit any significant activity up to 125 μM (*p* > 0.05, Figure S1L). Interestingly, from 1 to 4 h post AMP treatment, no significant OD600 differences were observed compared with PBS control groups. Their differences, however, increased as we further incubated the peptides with bacteria, reaching peaks after approximately 8 h and nearly up to the plateaus at 14 h (Figure 4a,b). The differences gradually diminished as the incubation proceeded, and almost disappeared at the end of incubation, which may be caused by peptide degradation. In addition, their predicted 3D structures (Figure 4c,d) seem to be beneficial to transmembrane due to existence of α helixes.

### 2.5. Deep Investigations on Several Interesting AMPs

#### 2.5.1. Hemoglobin-Derived AMPs

**Figure 5:**
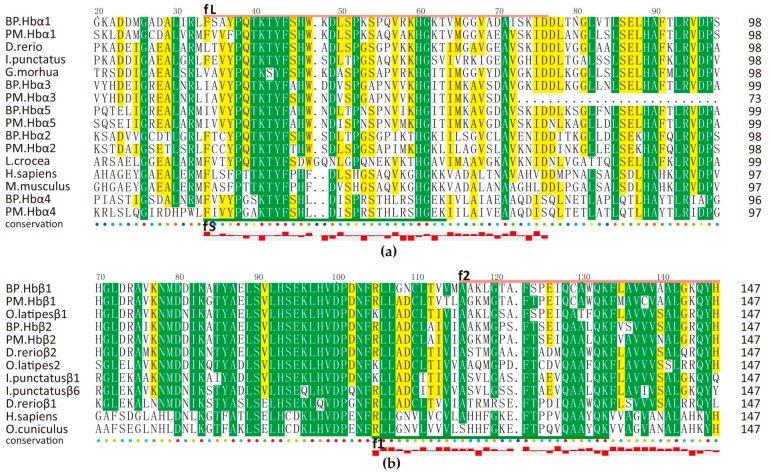
Multiple sequence alignment of Hbα (**a**) and Hbβ (**b**) from different species. fL/S and f1/2 refer to Long/Short fragment of Hbα and fragment 1/2 of Hbβ respectively. Yellow and green blocks mark the areas with sequence identity >50% and >80% respectively. Hydrophobicity is shown at the bottom of each alignment. Upper red box represents hydrophobic and underside box is hydrophilic.

Hemoglobins, composed of twin α and β globin subunits (α2β2), have broaden our views upon oxygen-transport proteins because of their immunological properties [31,32]. Many proteolytically generated hemoglobin-derived bioactive peptides have proven potent activities toward lots of pathogens [33,34,35,36]. We also predicted several antimicrobial peptides from each Hbα/β protein as shown in Figure 5, which was based on those previously reported active sequences (fL [33], fS [34], f1 [36], f2 [35]). Interestingly, Hb subunits from mudskippers all have more cysteines than those of other species. What is more, BP Hbβ1_1 (f1) was an active 29-aa peptide verified by our antimicrobial assay (Figure 4a).

The expression levels of Hbα1, α2, β1 were always the highest in gill of BP. However, it turned to liver in PM, as shown in Table 4. In addition, PM Hbβ2 was also detected by MS. It seems they have changeable peculiar function in PM. Except for their regular function, hemoglobin may mean a lot to host. Another three subunits were predicted using these fragments as query, we named α3, α4, α5 here temporarily. Then we employed BLAST in NCBI, subunits of BP were resembled with α subunits of other species while subunits of PM were similar to other types of subunit form of hemoglobin, namely epsilon (ε), zeta (ζ) etc., suggesting their diversification. Interestingly, they all locate next to previously predicted α/β subunit genes (Figure S2). Their gene structures are also the same as those highly expressed genes. However, they almost had no expression except that BP Hbα4 was detected by MS.

#### 2.5.2. Amyloid AMPs

Amyloid β-protein (Aβ) was firstly discovered with an antimicrobial activity against eight clinically relevant microorganisms, and it exhibits striking similarities to an archetypical human AMP LL-37 [37]. A previous report [38] compared activity of three typical human islet amyloid polypeptide (hIAPP, amylin), monomer β-sheet-rich fibrils, annular protofibrils (APFs), and portrayed their interaction with lipid membranes. It also pointed out that the antimicrobial activity of each aggregate is associated with the ability to induce membrane disruption.

**Figure 6:**
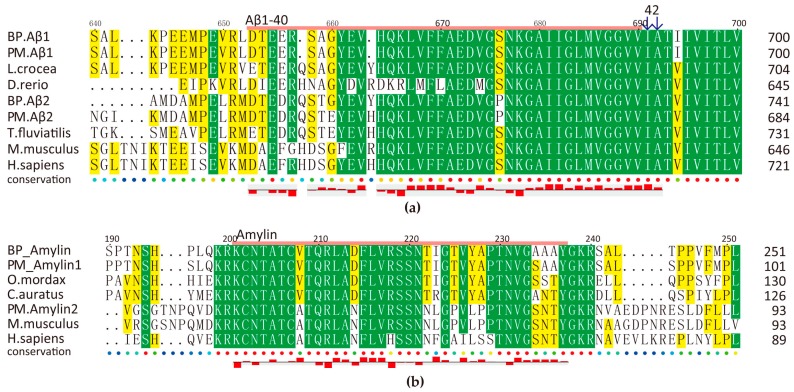
Multiple sequence alignment of Aβ (**a**) and amylin (**b**). Predicted AMPs are marked by pink line on the top of the sequences and their hydrophobicity are shown at the bottom. Yellow and green blocks indicate the areas with sequence identity >50% and >80% respectively.

We observed that this family is rather conserved among animal species (Figure 6). Intriguingly, amylin is cationic but Aβ peptides are anionic. Predicted Aβ1-40 and Aβ1-42 have a total net charge of −3 and −4 respectively, while the total net charge of amylin is +2 (BP) and +3 (PM). Related 3D predictions (data not shown) also suggest their potential antimicrobial roles as other reported members.

Amyloid genes of BP and PM have the same transcription patterns in five tissues although PM has much higher values, especially for Aβ2 in the brain and the skin (Table S4). Our antimicrobial assays have confirmed that BP amylin, a 37-aa peptide, presented a significant inhibition activity against *M. luteus* (Figure 4b). However, BP amylin is transcribed at a relative low level, and there are two types of amylin in PM (Figure 6b). Interesting, these peptides are rich in disulfide and α/β fold, which are related to their antimicrobial function.

What is more, some members of BPTI family (which is thought to have novel antifungal activity by inhibition of magnesium uptake [39]) are encoded by alternatively-spliced forms of Alzheimer’s Aβ precursors. In our present multiple sequence alignment (File S3), we observed that the area 295–352 of Aβ1 is the same as the peptide fragment of BPTI16 and BPTI20 in BP and PM respectively. The region 381–460 of BP Aβ2 is also the same as the fragment BPTI5 in BP, while it is exactly the missing piece of Aβ precursors in some species, including PM, zebrafish and rat. The relationship between Aβ peptides and BPTI is worth to be further studied.

#### 2.5.3. Piscidin Family

**Figure 7:**
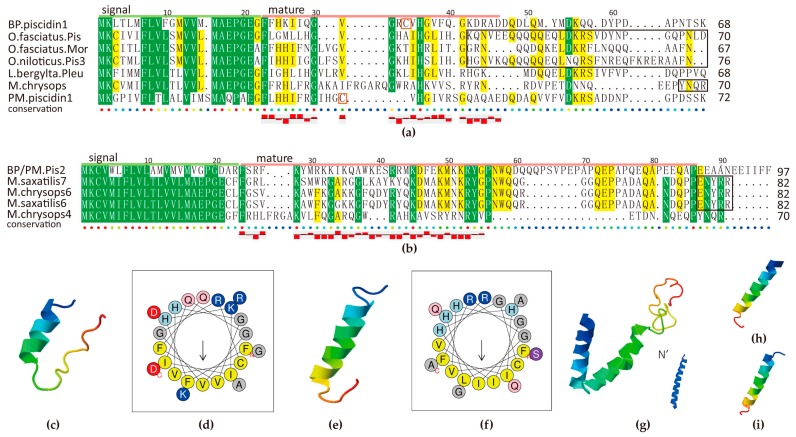
Multiple sequence alignment of piscidin1 (**a**) and piscidin2 (**b**) and related structure analysis of mature peptides (**c**–**i**). The well-known prodomains are outlined in the boxes of (**a**,**b**). Yellow and green blocks indicate the areas with sequence identity >50% and >80% respectively. 3D structure predictions of BP piscidin1 (**c**), PM piscidin1 (**e**), piscidin2 and its N terminal (**g**), misgurin1 (**h**) and misgurin2 (**i**) all possess a α-helix. Helical wheel diagrams of BP piscidin1 (**d**) and PM piscidin1 (**f**) suggest an amphipathic nature by the alignment of the hydrophobic residues along one side of the helix and the other side of hydrophilic residues.

Piscidin is known to be widespread among Perciformes fish. A growing number of evidence indicates that piscidin is an AMP gene containing multiple isoforms, while some of them are pseudogenes [40]. At first, we identified one piscidin homolog in each mudskipper. Their signal peptide revealed that they both belong to the antimicrobial 12 superfamily, according to the BLASTP results in NCBI. The highest ranked hit is the *Oplegnathus fasciatus* piscidin. BP piscidin1, with a 68-aa pre-propeptide, shares 47% identity with it; while PM piscidin1, with a length of 72 aa, hits it with 42% identity. They also closely hit piscidin3, moronecidin and pleurocidin-like family (Figure 7a).

As we know, many residues in the signal peptide region are tightly conserved in members of the piscidin family from fish of Acanthopterygii superorder, but rather low in mature peptides. We identified a new piscidin gene in the BP genome, which is located right next to the piscidin1. Hence we named it as piscidin2 here. Interestingly, the BP piscidin2, with a 294-nt open reading frame (ORF), presents 43% identity to *Morone saxatilis* class III piscidin7 (Figure 7b). We performed subsequent RT-PCR and confirmed that the same transcript exists in the PM genome. We speculate that the piscidin2 is a typical unique gene in mudskippers, which is worth further exploration. However, no other piscidin isoforms were identified in the mudskippers.

Several previously identified piscidin genes encode a precursor peptide, comprising a signal peptide of 22 residues that is removed by signal peptidase, a mature peptide of 22–25 residues, and a prodomain with variable length at the C-terminal region (marked in the black boxes of Figure 7a,b). Our predicted mature peptides of piscidin1 are rich in H and G, same as other piscidins, while piscidin2 is abound with R, K and Q. In addition, two piscidin1s contain a Cys in the mature region while seldom seen in others. The observation may unveil some mysteries of positive selection [41].

Two piscidin1s are predicted with an amphipathic α-helical conformation (Figure 7c–f) at high isoelectric point, which is beneficial to transmembrane activity and their combination with membrane, although they varied a lot between BP and PM compared with other piscidins. Piscidin2 is also with a perfect α-helical structure (Figure 7g), however, its mature peptide is longer (54 resideues) than other previously described piscidin isoforms (22 and 44–46 residues) [40]. Further investigation will be required to fully characterize their biological functions, especially their connection with particular characteristics of sequences, and confirmation of the designation. Interestingly, the predicted structures of misgurins in two species (Figure 7h,i) are of high similarity to piscidins, which are hence thought to be evolutionary related linear AMP family [20].

Our RPKM data (Table S4) demonstrated that both piscidin genes of BP are highly transcribed in the gill, followed by the skin, suggesting their potential important role against external pathogens. Further transcription profile is necessary to confirm their roles in different tissues, as piscidins are quite ubiquitously distributed in the hybrid striped bass [42].

Collectively, these data presented in the current study suggest that piscidin1 and piscidin2 of BP and PM are four putative novel members of fish piscidin family, which will advance our knowledge of the fish piscidin family and the contribution of piscidin to the mudskipper immunity for terrestrial adaptation.

## 3. Discussion

Mudskippers are characterized by their amphibious habitats, however few systematic studies on their components of innate system were reported. Here, for the first time, we identified 507 AMPs in two representative mudskipper species (BP and PM), and analyzed some highly transcribed AMP genes for tissue distribution differences between BP and PM. Compared with the results identified in other species (unpublished data), differences in number of AMPs and tissue distribution were observed. The molecular basis and evolutionary mechanism in amphibious organisms acclimating to various environment orchestrate the activity of massive immune substances, which are clarified scarcely. Our primary data of AMPs reported here, which is the most abundant in every individual species so far, provides valuable resources for illustrating them.

In the AMP catalogue of nine Chinese odorous frogs, 728 AMPs were identified using a genomic approach and 80 AMPs were confirmed by the peptidomic analysis [43]. This large-scale work also reported that identical AMPs were widely distributed in different frog species. Our current results revealed that 30 classified families do exist in the two mudskippers, in which 48 AMPs are overlapped in the two species, and 94 predicted precursors are confirmed by MS. Although post-translational modification of AMPs seldom happened in odorous frogs [43], while oxidation, deamidation and carbamidomethyl were detected in our MS results. Another de novo sequencing of Asian frog skin by tandem MS identified eight intact AMPs, and also revealed that cleavages located at C-S bonds on oxidized Cys [44]. We believe that chemical modification has built a link between the structures and desired functions [45,46,47]. Based on transcriptome data of skin in seven anurans, a total of 108 AMPs was reported [48]; among them, pancreatic neuropeptide YY and serpin are also confirmed in our mudskipper results.

Skin of amphibious creatures is always seen as the most valuable resource for AMP research [49]. Not only skin has been widely employed as the typical tissue for AMP identification, recently fish skeletal muscle is also noted as an immunologically active organ during pathogen infection [50]. Our RPKM value ranking (Table 2 and Table 4) revealed most highly expressed genes in skin also took a place in the highest value of muscle.

Popularly studied AMP families with their own genes, namely hepcidin, LEAP2 and defensin, are well confirmed in this study. They are all disulfide-bridged motifs contained AMPs and seem to be rather conserved among species [51,52,53]. Hepcidin, previously called LEAP1, plays not only directly against pathogens, but also as a key regulator of iron homeostasis by internalization and degradation, which is affected by inflammation or infection. Interestingly, transferrin, hemoglobin and GAPDH are also associated with Fe^2+^ ion [54,55,56]. T Leukocyte secreted components are also critical molecules against microbes, including defensin, chemokine, lactoferrin, lysozyme, BPTI, PLA2 (phospholipase A2), cathelicidin and ubiquitin [57]. Cathelicidin is a huge and ancient AMPs family in mammals and fish, however, we were not able to find a typical member in our databases. In addition, those released histones are known as mediators of thrombosis while histone and thrombin derived peptides are both microbicidal [58,59,60], bridging the immune system to coagulation system. It seems that these conserved multifunctional molecules have their own peculiar way of action among species. The relevance of their immune physiological reactions towards fighting against exogenous pathogens with regular pathways is worth further investigation.

The heredity diversity of AMPs gives us a chance to predict more reliable natural resources. When we run TBLASTN in our analysis pipeline to look for distant homologs, a relatively high e-value is considered to be useful for reporting results, such as 1e−1 or even 1 depending on the conservation of AMPs as it always need to take into account what you are looking for. We found out another three subunits of hemoglobin when we set cut-off with 1e−1. Same situation happened to piscidin (query 1649 and 2675), which is characterized by a highly divergent mature peptide but a highly conserved signal peptide. In addition, it should be noted that for smaller sequences (<30 nt), there is always a higher likelihood of random matches. In such cases, it is practical to relax the e-value cutoff. When we carried out alignment using two sequences only differed from addition of several amino acids in the C-terminal (such as Hbα derived AMPs 1339 and 2705), the longer is easier for identification. Considering all these factors, we could collect as many complete precursor sequences as possible. In this present study, we applied a relatively smaller e-value (1e−5) to predict sequences with comparatively high conservation for the sake of quality in alignment. Maybe we have missed out some AMPs, but it is believed that these authentic results are enough for us to investigate these important genes in amphibious mudskippers. In addition, our screening strategy is based on the known AMP databases; hence, it is indispensable to obtain more natural AMP templates with the traditional isolation and purification methods.

Although we have predicted so many potential AMPs, it is still hard to assess their activities, especially for those highly interconnected AMPs in complex environments. For example, β2M inhibited bacteria growth only in the presence of potassium [61]. Peptide degradation, structural folding in membranes and oligomerization should also be considered [62]. Meanwhile some peptides with one amino acid substitution from known active AMPs are likely to exhibit a different activity, such as our tested ERH and misgurin [22,24]. In addition, it is hard to synthesize a peptide chemically with disulfide-bridges, not to mention their modifications. Hence designing engineering of many peptides devoted much work on developing efficacious AMPs [63,64,65,66]. Moreover, antimicrobial activity of any AMP appears to depend on the specifically targeted microorganisms [67], and most AMPs play vital immune-regulative effects and synergistical actions with other antimicrobial molecules [68,69,70,71]. From the perspective of evolution, positive selection has occurred to many AMPs genes among various species [72,73]. Hence, potential coevolution with surrounding microorganisms or connection with geographical distribution is worth deep exploration.

## 4. Materials and Methods

### 4.1. Data Collection

All the 2705 amino acid sequences with previously validated antimicrobial activity were retrieved from online Antimicrobial Peptides Database (APD3, http://aps.unmc.edu/AP/main.php), National Center for Biotechnology Information (NCBI) database (https://www.ncbi.nlm.nih.gov/) and PubMed (https://www.ncbi.nlm.nih.gov/pubmed/) (Table S1). Subsequently we constructed a local reference AMP database to search against our previously established seven datasets, including assembled whole-genomes with high quality which can be used for genome-wide comparison, annotated gene sets and assembled transcriptomes with transcription patterns in multiple tissues (brain, gill, liver, muscle, skin) of BP and PM. More details of these data can be obtained from our previous report of mudskipper genomes [21].

### 4.2. Prediction and Identification of AMPs

We applied standard homology searches to predict AMPs. Firstly, we built an index database for each dataset by running a formatdb command (Standalone BLAST for UNIX). We subsequently employed TBLASTN (e-value: 1e−5) to run reference AMPs against the seven datasets respectively, followed by in-house script to deal with these results, and filtered those hits with query align ratio less than 0.5. We also used an in-house pipeline to combine targeting information of hit peptides and extracted their corresponding homology coding DNA sequences. All these predicted sequences were further manually inspected using BioEdit tool [74] in each different group. Those partial or prematurely incomplete transcripts and genes were removed.

Furthermore, we employed multiple sequences alignments by mafft-7.037 [75] to align these putative AMPs with other known proteins and sorted them by mutual similarity. Amino acids that are conserved in the same position were shaded by TexShade [76].

### 4.3. MS Analysis and Protein Identification

Samples of skin and muscle were used to extract proteins from BP and PM. Isolated samples were homogenized in tissue lysis buffer (Pierce Biotechnology, Rockford, IL, USA) supplemented with 1 mM phenylmethanesulfonyl fluoride (PMSF) and 2 mM ethylenediaminetetraacetic acid (EDTA) in centrifuge tubes. The mixtures were placed still for 5 min after vortex oscillation and addition of 10 mM dithiothreitol (DTT), and then spun at 25,000× *g* for 20 min at 4 °C to obtain supernatants. Proteins in the supernatants were denatured with 8 M urea in 0.1 M Tris-HCl, pH 8.5 and reduced with 10 mM DTT at 56 °C for 1 h. After cooled down to the room temperature, the proteins were alkylated with 55 mM Iodoacetamide (IAM) in the dark at room temperature for 45 min. Four volumes of cool acetone was added multiple times for 2 h at −20 °C until the supernatants became colorless. After centrifuged for 20 min, the supernatants were aborted. Finally, the supernatants were collected after spinning at 25,000× *g* for 20 min at 4 °C with addition of the tissue lysis buffer. The protein concentrations were measured with the Bradford reagent and BSA (Sigma, St. Louis, MO, USA) as reported in a standard protocol [77]. Each sample was enzymatically dissociated by Trypsin for 12 h. The alkylated protein solution was diluted with 8 M urea to 1 mL, and then fractionated in a Strata-X C18 column (Phenomenex, Torrance, CA, USA), which had previously been conditioned with methanol. After loading the protein solution, the column was washed with 0.1% formic acid (FA) in 5% acetonitrile (ACN) and eluted with 80% ACN. The eluates were dried in a SCANVAC concentrator (LaboGene, Lynge, Denmark) and then stored at 20 °C for further analysis.

After dissolved in 0.1% FA and 2% ACN, the peptides were separated by a Nanodrop system Eksigent415 (SCIEX, Framingham, MA, USA). High Performance Liquid chromatography (HPLC) with tandem MS (LC-MS/MS) was performed on a prominence nano-HPLC system coupled with Triple TOF 5600 (SCIEX, Framingham, MA, USA). The mass spectrometers were operated in a data-dependent mode, automatically switching between MS and MS2 acquisition. Survey of full scan MS spectra (*m*/*z* 350–1500) was acquired with a resolution of 40,000. The 30 most intense ions were sequentially isolated and fragmented by high energy collisional dissociation (HCD). Peptides with unassigned charge states as well as with charge states less than +2 or more than +6 were excluded from fragmentation. Fragment spectra were recorded in the TOF mass analyzer (SCIEX, Framingham, MA, USA). The dynamic exclusion was enabled with repeat count 2 and exclusion duration of 8 s, and quality control was used to ensure the accuracy of acquired data.

For peptide identification, MS/MS data were searched against our established local reference AMP database (Table S1) using Mascot 2.3.02 and Percolator [78]. Peptide and fragment mass tolerance was set as 0.05 and 0.1 Da respectively. PSM-level (FDR < 0.01) and protein-level (FAR < 0.01) were used for data analysis. All the procedures were followed by the standard commercial pipeline of BGI-MSI (BGI, Shenzhen, China).

### 4.4. Synthesis of Custom Peptides

All predicted active fragments were synthesized via Fmoc chemistry and cleavage of the peptide from the resin [79]. After synthesis, each peptide was purified via analytical HPLC using a Kromasil 100-5-C18 column (4.6 mm × 250 mm, AkzoNobel, Bohus, Sweden), with an acetonitrile gradient that was eluted at 1 mL/min, where buffer A was 0.1% trifluoroacetic in 100% acetonitrile and buffer B was 0.1% trifluoroacetic in 100% water. The peptides with a purity of >90% were generated at GL Biochem Ltd., (Shanghai, China). Purity of the synthesized peptide was confirmed by HPLC-MS/MS. These synthesized AMP peptides were finally reconstituted in sterile deionized water and stored at −80 °C before use.

### 4.5. Antibacterial Assays

Antimicrobial activities of 23 synthesized AMPs against *Aeromonas hydrophila*, *Vibrio parahaemolyticus*, *Staphylococcus aureus*, *Micrococcus luteus* were examined as previously reported [80] with some modifications. Firstly, microorganisms in a logarithmic phase were centrifuged, washed by PBS (phosphate buffered saline) for three times, and then resuspended in PBS (104 CFU/mL). Subsequently, 10 μL of PBS dissolved peptides were mixed with 10 μL of bacterial suspensions respectively for an incubation at room temperature for 1 h. PBS was used as the blank control. Finally, the mixtures were dispensed into a 96-well plate containing 200 μL of LB medium. In a preliminary screening, the final concentration of all samples was 250 μg/mL. In the determination of minimum inhibitory concentration (MIC) of three peptides, serial dilutions of peptides (7.8 to 250 μg/mL) were added. Plates were then placed in a microplate reader (Biotek, Winooski, VT, USA) at 28 °C (for G− bacteria) or 37 °C (for G+ bacteria) with a shake every five seconds. OD600 values were measured every 0.5 h for of a total of 16 h to generate the growth curves. Experiments were repeated three times for each peptide.

All the data was presented as mean ± standard deviation (*n* = 3), and analyzed with Statistical Package for Social Sciences (SPSS, IBM, Armonk, NY, USA) 16.0 and Origin (OriginLab, Northampton, MA, USA). The significant differences among groups were tested by paired two-tailed Student’s *t*-test and multiple comparisons. Statistically significant differences were designated at *p* < 0.05 and extremely significant at *p* < 0.01.

### 4.6. Computational Molecular Profiling of AMPs

Composition analysis of peptides, net charge at neutral pH and isoelectric point were determined by employing the pepstats software of EMBOSS-6.6.0 [81]. The Shiffere-Edmundson helical wheel diagrams, the hydrophobicity and the hydrophobic moment were predicted using HeliQuest from http://expasy.org/tools [82]. Tertiary structure of each AMP was predicted using the I-TASSER server [83].

## 5. Conclusions

We provide a high-throughput strategy to discover potential novel AMPs by using a combination of genomic, transcriptome and peptidomic datasets. Compared with the traditional chromatograph desired to isolate small molecules, our present work accelerates the studies on differences of innate immune system between species and established a large template database for screening novel AMPs with a powerful capacity. Out of the newly identified 507 AMPs with classification into 30 families, 449 are novel and 94 precursors are confirmed at the protein level. Numerous mRNA profiling differences between the two representative mudskippers with different ecological habits may suggest specialized contribution of these examined AMPs.

Present findings suggest that PM has more extreme AMP diversity than BP in nature. Here we also pointed out the importance of two highly active AMP families, hemoglobin and amyloid, in fighting against exogenous pathogens. What is more, to our knowledge, piscidin family seems presumed to be unique in mudskippers compared with other members in different species. We are going to explore detailed molecular mechanisms of some typical AMP types among species in future studies, and unravel the self-adaptive mechanisms of related organisms. These data will surely provide valuable resources for further development of marine drugs, particularly those highly active peptides will become a treasure house for novel drug discovery.

## Figures and Tables

**Table 1:** Identified putative AMP numbers from seven genomic and transcriptomic datasets.

Species	Scaffold	Gene Sets	Brain	Gill	Liver	Muscle	Skin
BP	98	239	131	174	118	107	103
PM	126	225	145	165	144	103	156

**Table 2:** The top 20 RPKM ranking of individual AMPs within each of the four transcriptome datasets.

Ranking	BP Gill	BP Skin	PM Gill	PM Skin
1	Hbα2	Hbα2	β2M1	GAPDH1
2	Hbβ1	Hbβ1	CCL4	Ubq/poly1
3	Hbα1	Hbα1	Hbα2	β2M1
4	Piscidin2	GAPDH1	CXCL8	Ubq/RPL40e
5	Ubiquicidin	Ubiquicidin	Ubq/poly1	Hbα2
6	Ubq/RPL40e	Ubq/RPL40e	Ubq/RPL40e	Hbβ1
7	GAPDH3	CCL1	Hbβ1	Ubq/RPS27a
8	DWD2	GAPDH3	CCL2_2	GAPDH6
9	Thrombin8	DWD2	Ubq/RPS27a	Misgurin2
10	Defensinα2	CCL3	Hbα1	Hbα1
11	CCL1	Saposin2	GAPDH1	SK84_2
12	Piscidin1	H2A5	SK84_2	SK84_1
13	Saposin2	CCL2	CCL2	Serotransferrin
14	CXCL7	BPTI6	Thrombin40	CXCL8
15	CCL3	Hepcidin2	CXCL7	Saposin2
16	GAPDH1	SWD3	GAPDH6	Ubiquicidin
17	CCL2	RNase6	CCL3	BPTI18
18	Defensinα1	Glyrichin	Saposin2	BPTI24
19	SWD3	CXCL7	Thrombin9	Ap
20	Glyrichin	CCL4	H2A1	CcAMP

**Table 3:** Statistics of the MS results.

Sample	Total/Identified Spectra	AMP Spectra	Identified Peptides/Proteins	AMP Peptides/Proteins
BP Skin	168,975/65,778	3843	10,985/1292	308/45
BP Muscle	137,162/49,488	1313	5140/435	124/17
PM Skin	166,870/68,187	4013	9898/1442	270/47
PM Muscle	144,915/52,062	1417	4787/577	155/22

**Table 4:** RPKM values of the Hb subunits in five tissues of BP and PM.

Tissue	Hbβ1	Hbβ2	Hbα1	Hbα2	Hbα3	Hbα4	Hbα5
BP Brain	3691.38	2.72	2909.06	16,212.60	0.20	4.82	0.44
PM Brain	128.06	0	63.62	230.17	0	0	0
BP Gill	12,092.00	7.21	6047.51	41,419.70	0	10.05	0.04
PM Gill	1652.96	0	768.13	2928.19	0	0.06	0
BP Liver	1767.70	0.19	440.72	5545.23	0	2.99	0
PM Liver	4448.83	0.24	1422.59	8908.93	0	0.09	0
BP Muscle	4927.71	0.65	2460.18	14,000.1	0	1.75	0.06
PM Muscle	919.64	0	339.07	1678.62	0	0.08	0
BP Skin	7408.15	4.75	4211.22	17,282.30	0.57	5.31	0
PM Skin	996.73	0	354.46	1529.95	0	0.10	0

## Supplementary Materials

The following are available online at www.mdpi.com/1660-3397/15/11/364/s1, Figure S1: Preliminary antimicrobial activity screening of AMPs; Figure S2: Localization of Hb genes on the genomes of BP and PM; Table S1: Collection of reported AMPs; Table S2: Alignment results from the genome datasets; Table S3: Alignment results from the transcriptome datasets; Table S4: Predicted AMP genes and their RPKM values; Table S5: Detected fragments of AMP precursors by MS; Table S6: AMPs examined by the antimicrobial assays and sequences composition analysis; File S1: Established AMP database from the two mudskippers; File S2: GO enrichment of genes identified from the skin and muscle of BP and PM by a MS analysis; File S3: Multiple sequence alignment of amyloid precursors in various species.
