# Supplementary material for: High-Throughput Identification of Antimicrobial Peptides from Amphibious Mudskippers
Source: Mar Drugs. 2017 Nov 22;15(11):364. doi: 10.3390/md15110364 (PMC5706053; doi:10.3390/md15110364)
Supplement: Supplementary file 1 [file marinedrugs-15-00364-s001.zip › Supplementary/File S2.pdf]

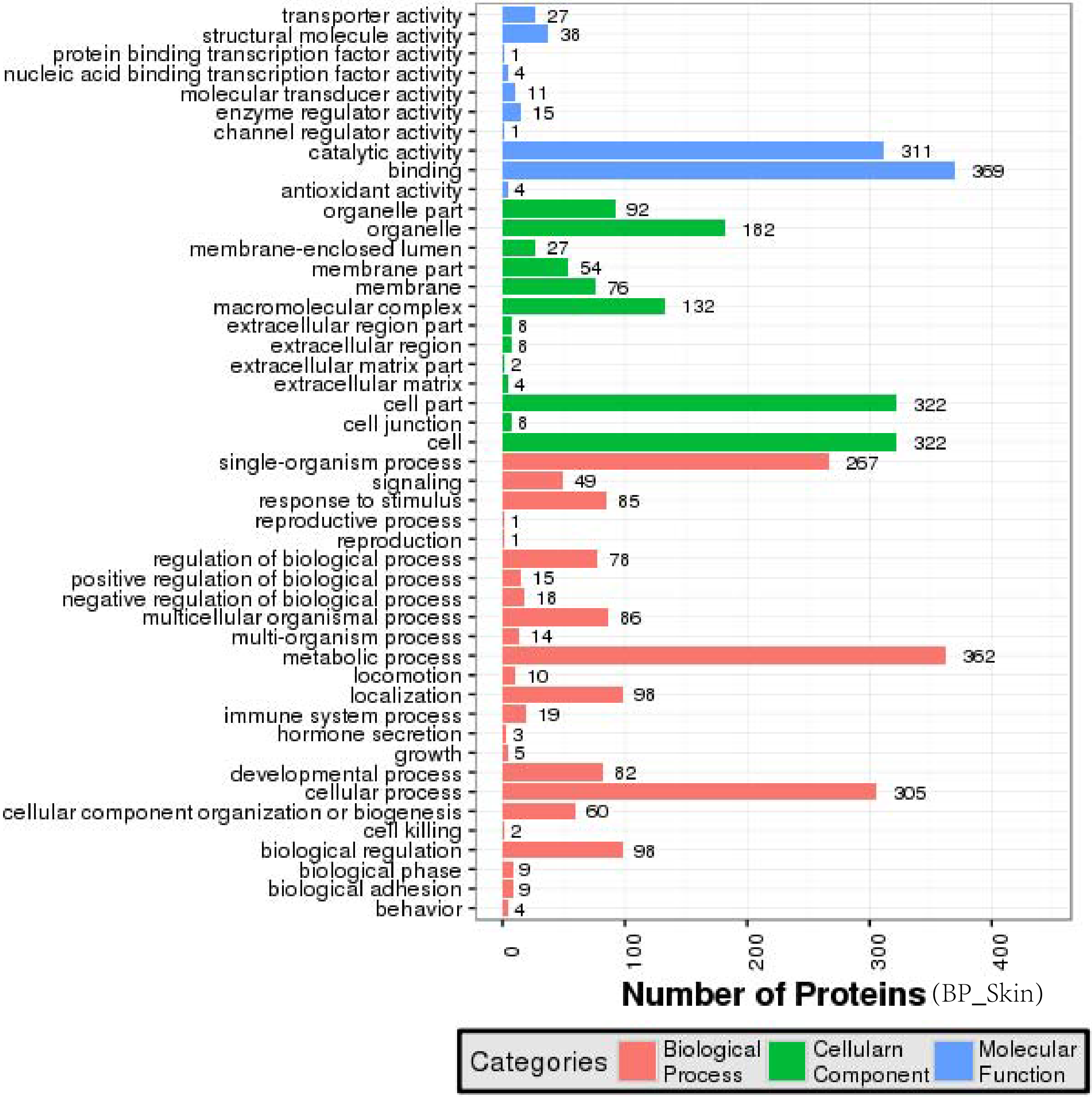
transporter activity
structural molecule activity
protein binding transcription factor activity
nucleic acid binding transcription factor activity
molecular transducer activity
enzyme regulator activity
channel regulator activity
catalytic activity
binding
antioxidant activity
organelle part
organelle
membrane-enclosed lumen
membrane part
membrane
macromolecular complex
extracellular region part
extracellular region
extracellular matrix part
extracellular matrix
cell part
cell junction
cell
single-organism process
signaling
response to stimulus
reproductive process
reproduction
regulation of biological process
positive regulation of biological process
negative regulation of biological process
multicellular organismal process
multi-organism process
metabolic process
locomotion
localization
immune system process
hormone secretion
growth
developmental process
cellular process
cellular component organization or biogenesis
cell killing
biological regulation
biological phase
biological adhesion
behavior
27
38
1
4
11
15
1
311
369
4
92
182
27
54
76
132
8
8
2
4
322
8
322
267
49
85
1
1
78
15
18
86
14
362
10
98
19
3
5
82
305
60
2
98
9
9
4
0
100
200
300
400
Number of Proteins (BP_Skin)
Categories
Biological Process
Cellularn Component
Molecular Function

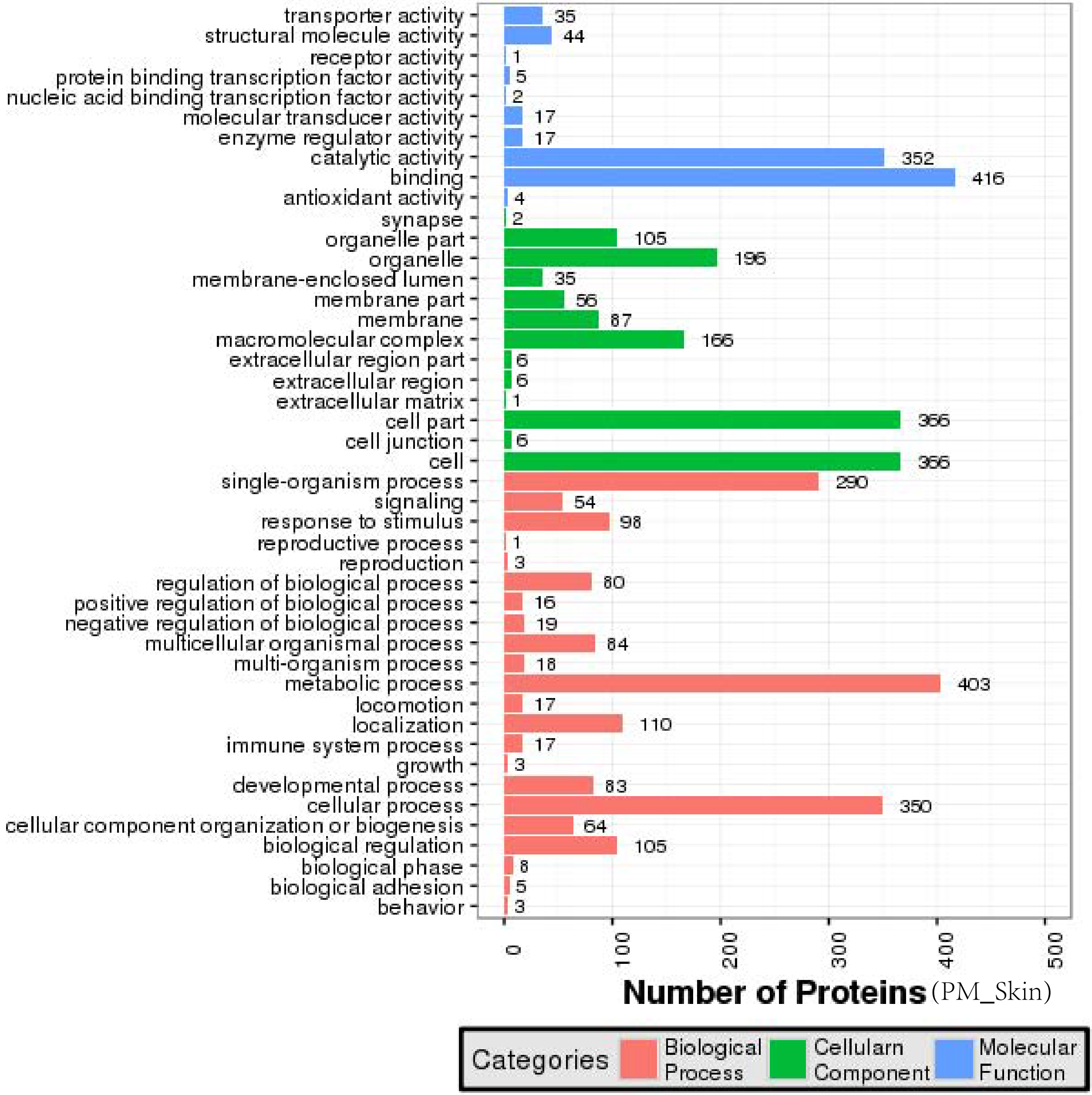

transporter activity 35
structural molecule activity 44
receptor activity 1
protein binding transcription factor activity 5
nucleic acid binding transcription factor activity 2
molecular transducer activity 17
enzyme regulator activity 17
catalytic activity 352
binding 416
antioxidant activity 4
synapse 2
organelle part 105
organelle 196
membrane-enclosed lumen 35
membrane part 56
membrane 87
macromolecular complex 166
extracellular region part 6
extracellular region 6
extracellular matrix 1
cell part 366
cell junction 6
cell 366
single-organism process 290
signaling 54
response to stimulus 98
reproductive process 1
reproduction 3
regulation of biological process 80
positive regulation of biological process 16
negative regulation of biological process 19
multicellular organismal process 84
multi-organism process 18
metabolic process 403
locomotion 17
localization 110
immune system process 17
growth 3
developmental process 83
cellular process 350
cellular component organization or biogenesis 64
biological regulation 105
biological phase 8
biological adhesion 5
behavior 3
0
100
200
300
400
500
Number of Proteins (PM_Skin)
Categories
Biological Process
Cellularn Component
Molecular Function

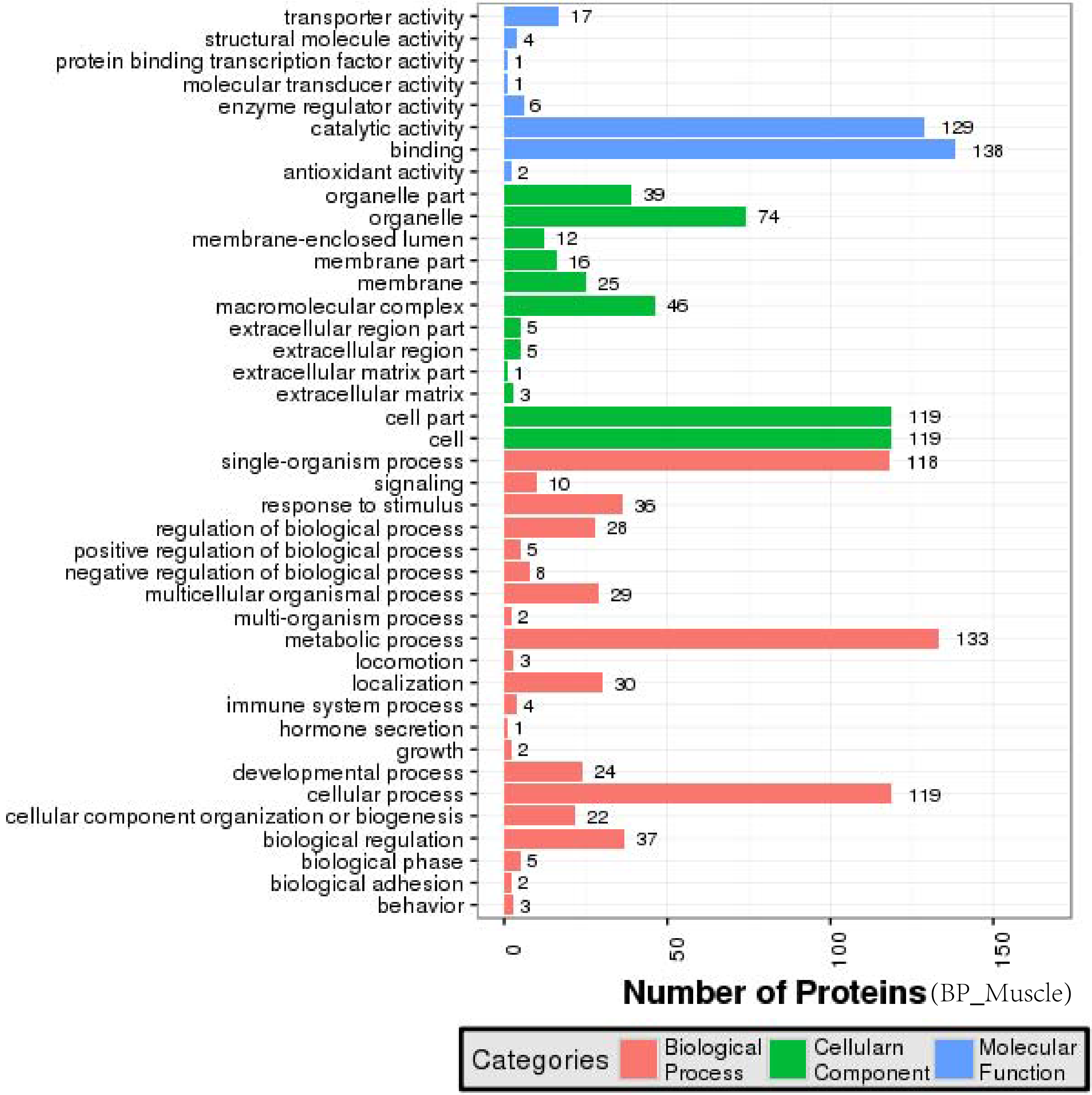
transporter activity
structural molecule activity
protein binding transcription factor activity
molecular transducer activity
enzyme regulator activity
catalytic activity
binding
antioxidant activity
organelle part
organelle
membrane-enclosed lumen
membrane part
membrane
macromolecular complex
extracellular region part
extracellular region
extracellular matrix part
extracellular matrix
cell part
cell
single-organism process
signaling
response to stimulus
regulation of biological process
positive regulation of biological process
negative regulation of biological process
multicellular organismal process
multi-organism process
metabolic process
locomotion
localization
immune system process
hormone secretion
growth
developmental process
cellular process
cellular component organization or biogenesis
biological regulation
biological phase
biological adhesion
behavior
17
4
1
1
6
129
138
2
39
74
12
16
25
46
5
5
1
3
119
119
118
10
36
28
5
8
29
2
133
3
30
4
1
2
24
119
22
37
5
2
3
0
50
100
150
Number of Proteins (BP_Muscle)
Categories
Biological Process
Cellularn Component
Molecular Function

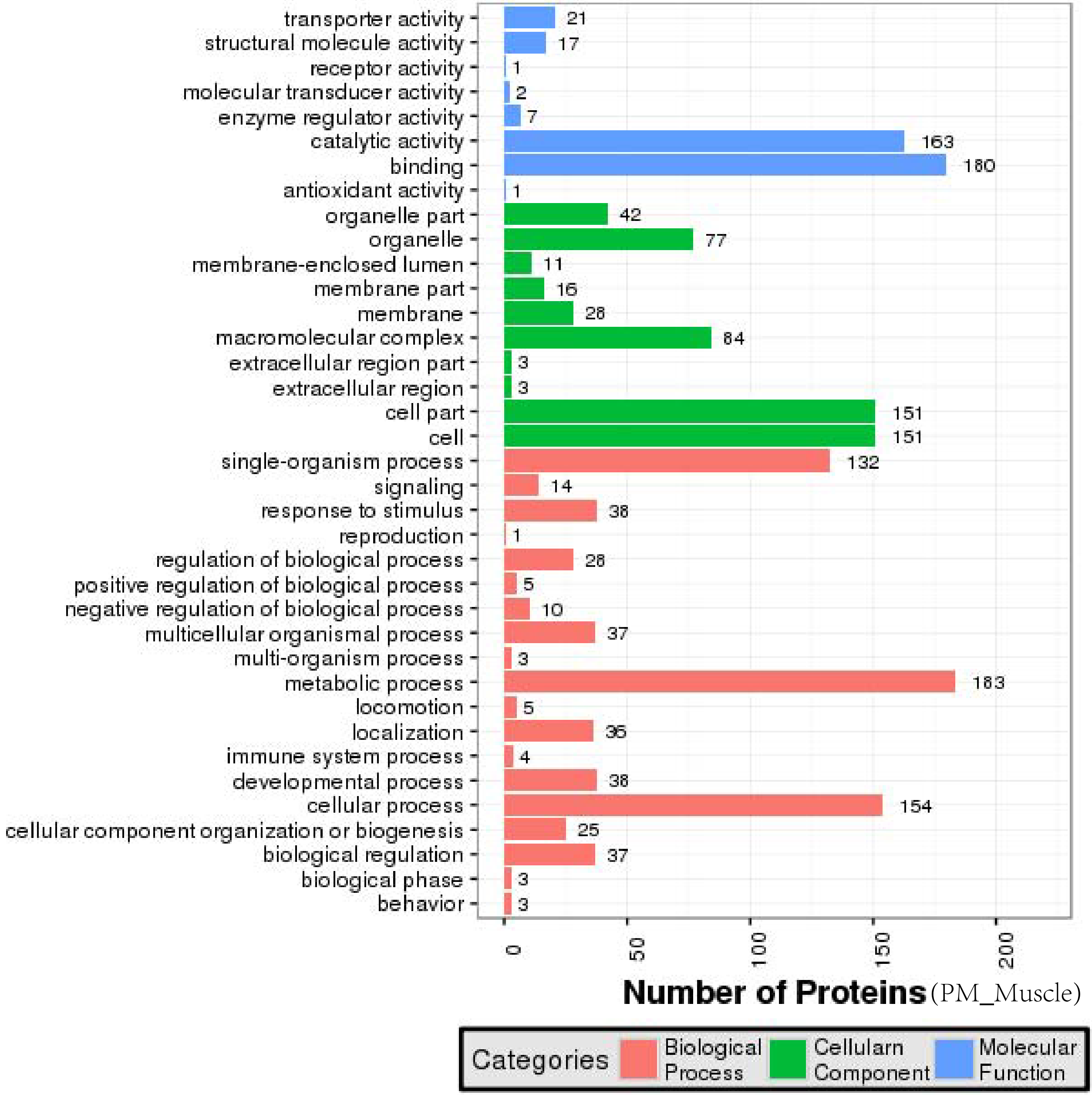

transporter activity 21
structural molecule activity 17
receptor activity 1
molecular transducer activity 2
enzyme regulator activity 7
catalytic activity 163
binding 180
antioxidant activity 1
organelle part 42
organelle 77
membrane-enclosed lumen 11
membrane part 16
membrane 28
macromolecular complex 84
extracellular region part 3
extracellular region 3
cell part 151
cell 151
single-organism process 132
signaling 14
response to stimulus 38
reproduction 1
regulation of biological process 28
positive regulation of biological process 5
negative regulation of biological process 10
multicellular organismal process 37
multi-organism process 3
metabolic process 183
locomotion 5
localization 36
immune system process 4
developmental process 38
cellular process 154
cellular component organization or biogenesis 25
biological regulation 37
biological phase 3
behavior 3
0
50
100
150
200
Number of Proteins (PM_Muscle)
Categories
Biological Process
Cellularn Component
Molecular Function
