# Supplementary figures and images for: High-Throughput Identification of Antimicrobial Peptides from Amphibious Mudskippers
Source: Mar Drugs. 2017 Nov 22;15(11):364. doi: 10.3390/md15110364 (PMC5706053; doi:10.3390/md15110364)

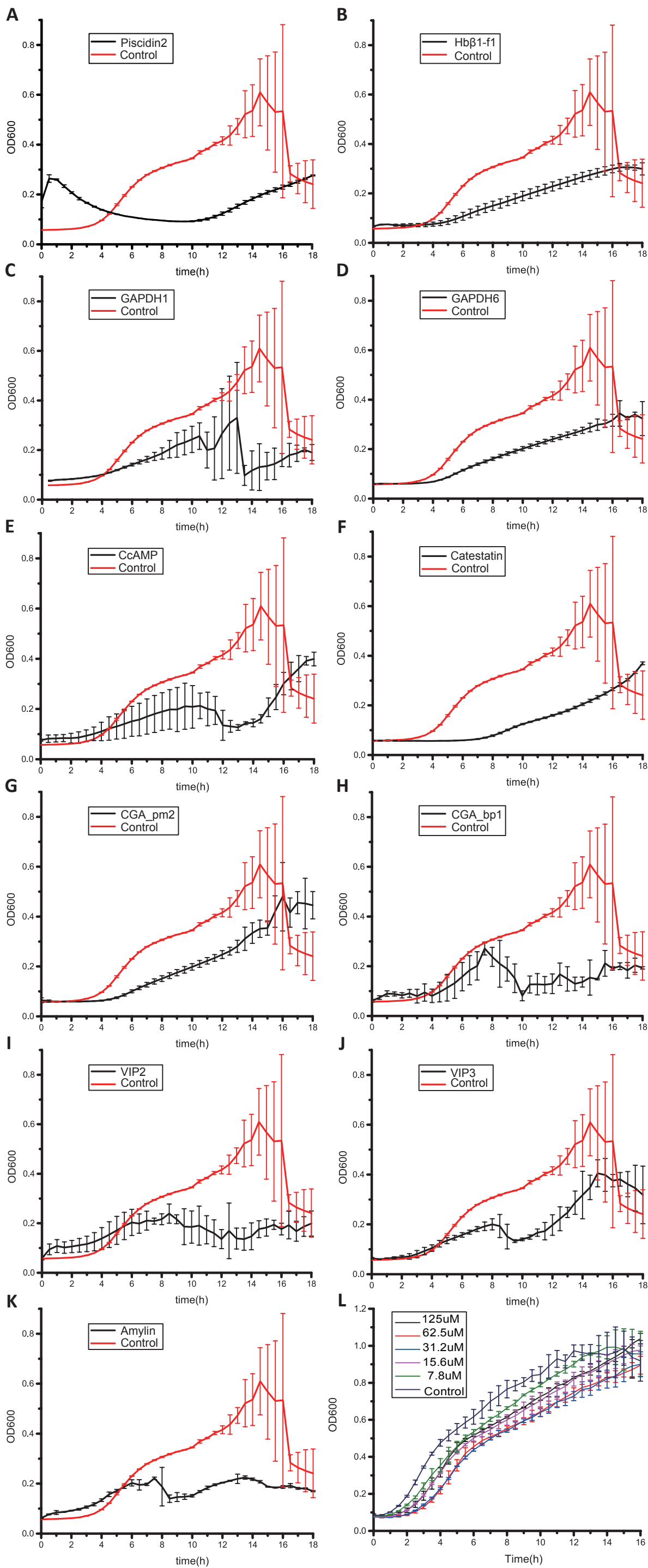

Supplement: Supplementary file 1 [file marinedrugs-15-00364-s001.zip › Supplementary/Figure S1.pdf]

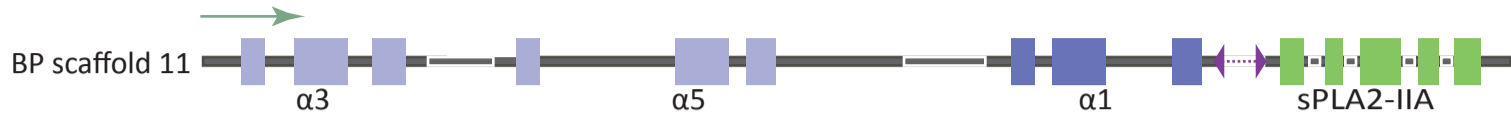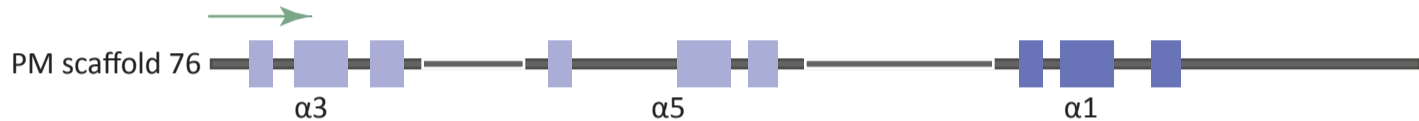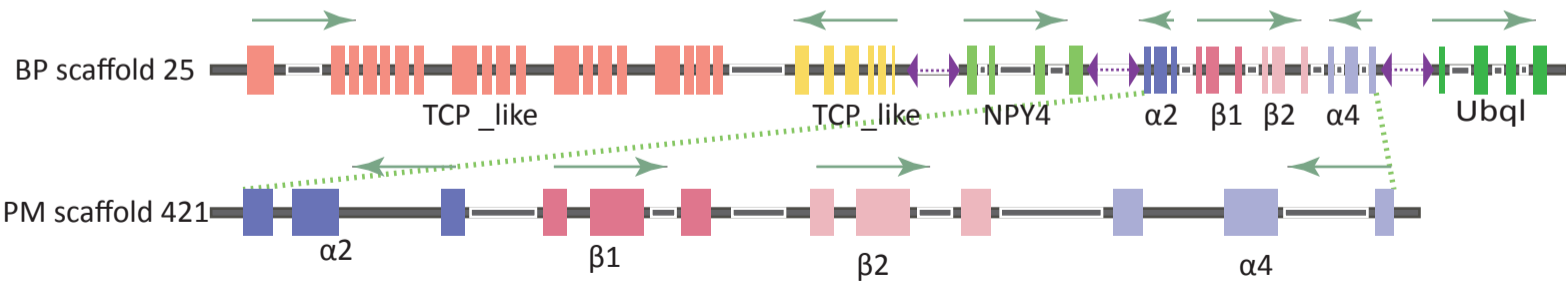

Supplement: Supplementary file 1 [file marinedrugs-15-00364-s001.zip › Supplementary/Figure S2.pdf]
